# Rethinking How National Health Programmes Are Taught: Team-Based Decision Learning Versus Mini-Interactive Lectures Among Undergraduate Medical Students

**DOI:** 10.7759/cureus.117822

**Published:** 2026-10-12

**Authors:** Minu Mohan, Diya T Jose, Binu Areekal

**Affiliations:** 1 Community Medicine, Government Medical College, Ernakulam, Ernakulam, IND

**Keywords:** active learning methods, competency-based medical education, health professions education, innovation in medical education, innovative teaching learning, teaching–learning methods in medicine, team-based learning (tbl), undergraduate medical students, undergraduate teaching

## Abstract

Purpose: To compare the learning outcomes, learner engagement, and student satisfaction associated with team-based decision learning (TBDL), an adaptation of team-based learning, and small group discussion supplemented by a mini-interactive lecture (SGD-MIL) for teaching National Health Programmes to Phase III Bachelor of Medicine and Bachelor of Surgery (MBBS) students.

Methods: A non-randomised quasi-experimental crossover study was conducted among 56 Phase III MBBS students at Government Medical College, Ernakulam, India across six National Health Programme topics. Students received TBDL for three topics and SGD-MIL for three other topics, with the teaching method reversed in the other posting batch. Immediate post-test scores were analysed topic-wise, and a linear mixed-effects model was used to estimate the overall effect of teaching method while accounting for differences across topics and repeated observations within students; teaching method and topic were included as fixed effects, with student specified as a random intercept. Short-term retention, learner engagement, and satisfaction were also assessed.

Results: TBDL produced higher immediate post-test scores for the National Tuberculosis Elimination Programme (NTEP), National AIDS Control Programme (NACP), and Reproductive and Child Health (RCH), but not for the National Programme for Prevention and Control of Non-Communicable Diseases (NP-NCD), National Vector Borne Disease Control Programme on Malaria (NVBDCP - Malaria), or National Vector Borne Disease Control Programme on Vector Borne Diseases Other Than Malaria (NVBDCP - Other VBDs). In a mixed-effects model using 296 scores from 55 students, the adjusted mean was 1.07 points higher with TBDL (95% CI: 0.53-1.61; P < 0.001), with a significant teaching method × topic interaction (P < 0.001). Short-term retention did not differ (P = 0.721), while TBDL was associated with higher engagement across all five domains.

Conclusion: TBDL improved immediate learning for selected National Health Programme topics and consistently enhanced learner engagement, while short-term retention was comparable between methods. These findings support TBDL as a complementary, decision-oriented strategy for selected community medicine topics, particularly where learners must apply programme knowledge to realistic implementation decisions.

## Introduction

With the adoption of the Competency-Based Medical Education (CBME) framework by the National Medical Commission (NMC) in 2019, undergraduate medical education in India has shifted from time-based didactic instruction towards outcome-oriented learning [1]. This transformation has necessitated a departure from conventional, lecture-heavy formats in favour of more learner-centric, small-group teaching modalities so that medical students are equipped with the requisite knowledge, skills, and attitudes to address real-world health problems beyond factual recall. This shift has important implications for the teaching of community medicine, particularly National Health Programmes (NHPs), where competence extends beyond recalling programme components. The current curriculum expects Indian medical graduates to understand the planning, implementation, monitoring, evaluation, surveillance, and management of NHPs, competencies that require contextual reasoning and decision-making. Hence, there is a need for learning environments in which students can apply programme knowledge to realistic public health problems that demand higher-order cognitive skills such as prioritisation, decision-making, contextual reasoning, and feasibility assessment [1].

Educational research on NHP teaching remains limited, and available studies have largely assessed knowledge, attitudes, or practices related to selected programmes rather than higher-order programme reasoning. A study done in South India in 2024 showed that 61.4% to 71.8% of the students had good to better knowledge of the basic concepts of national programmes related to tuberculosis, leprosy, and HIV, while a study done in North India showed a mean score of knowledge of 4.82 ± 1.75 and practice of 6.73 ± 2.47 [2,3]. Existing teaching approaches such as interactive lectures, seminars, small group discussions, and field exposure can support knowledge acquisition, but they may provide fewer structured opportunities for students to practise complex, programme-oriented decisions.

Team-based learning (TBL) can address this gap by combining preparation, readiness assurance, collaborative application, and faculty synthesis [4-7]. However, the full TBL format can be difficult to implement within routine two-hour community medicine teaching sessions. Team-based decision learning (TBDL) was therefore developed for this study as a time-feasible adaptation that retains the decision-making and collaborative application components most relevant to NHP teaching.

TBDL emphasises epidemiological reasoning, prioritisation of interventions, interpretation of programme indicators, and justification of implementation decisions within the limited time available for small group teaching. Mini-interactive lectures delivered within small group discussion (SGD-MIL) provide structured faculty guidance and focused explanation of core content. Although TBL has been widely studied, evidence on a decision-oriented adaptation for undergraduate NHP teaching in community medicine is limited; this study compared TBDL with SGD-MIL for immediate learning, short-term retention, learner engagement, and satisfaction. We hypothesised that TBDL would improve learning outcomes and produce higher engagement and satisfaction than SGD-MIL.

## Materials and methods

A non-randomised, quasi-experimental study employing a counterbalanced, crossover design was conducted among Phase III Bachelor of Medicine and Bachelor of Surgery (MBBS) students. The study was conducted in the Department of Community Medicine, Government Medical College, Ernakulam, India, during routine Phase III MBBS clinical postings from May 2026 to August 2026. As individual randomisation was not undertaken, two consecutive Phase III MBBS posting batches, each comprising 28 students, participated in the study. Each batch experienced both teaching methods, with the two methods assigned to different NHP topics in a counterbalanced sequence. The counterbalanced crossover design ensured that each posting batch was exposed to both teaching-learning methods while learning different NHP topics, thereby minimising the influence of inter-batch differences. Thus, the posting batch served as the unit of assignment, while student-level outcomes were analysed according to the teaching-learning method used for each topic. The students who attended the sessions and the corresponding assessments were eligible for inclusion in the specific outcome analysis. Students who were absent from a teaching session or the corresponding assessment had no data available for that specific outcome and were therefore excluded from that outcome analysis. They were not excluded from other analyses for which they had complete data. The overall sequence of participant enrolment, assignment to the two counterbalanced teaching sequences, exposure to both teaching methods, outcome assessment, and statistical analysis is presented in Figure 1. The study was reported in accordance with the principles of the Transparent Reporting of Evaluations with Non-randomised Designs (TREND) statement [8].

**Figure 1:**
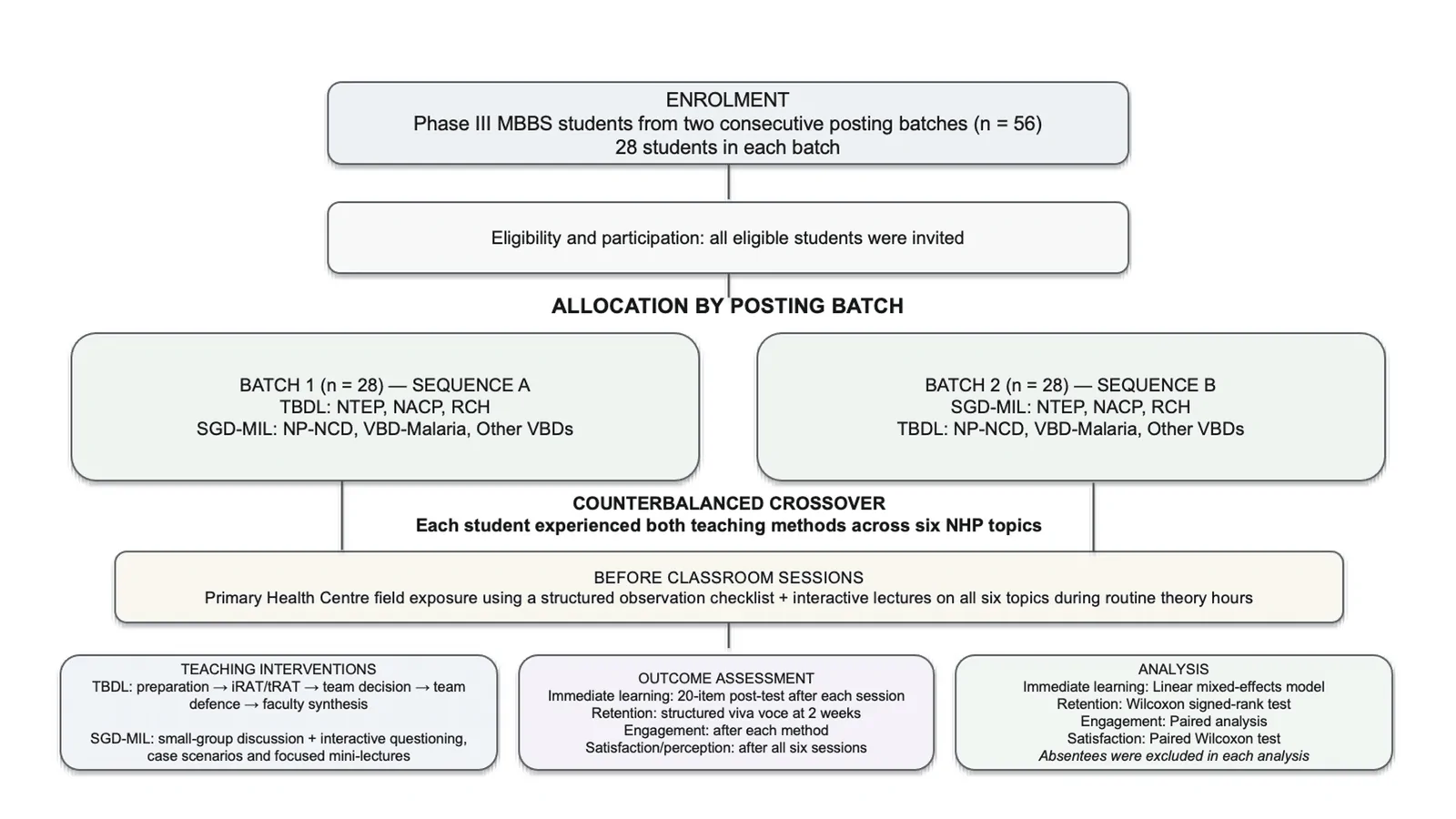
Study flow diagram of the non-randomized, quasi experimental study employing a counterbalanced crossover design. The study included 56 phase III MBBS students from two consecutive posting batches, with 28 students in each batch. The two batches followed opposite teaching sequences, allowing each student to experience both teaching methods across six National Health Programme topics. Immediate learning, retention, learner engagement, and satisfaction/perception were assessed at the specified time points. TBDL, team-based decision learning; SGD-MIL, small group discussion with mini-interactive lecture; NHP, National Health Programme; iRAT, individual readiness assurance test; tRAT, team readiness assurance test; NTEP, National Tuberculosis Elimination Programme; NACP, National AIDS Control Programme; RCH, Reproductive and Child Health; NP-NCD, National Programme for Prevention and Control of Non-Communicable Diseases; VBD, vector-borne diseases.

Selection of topics and assignment

The NHP topics included in the study were selected using a two-stage participatory process. Initially, a pile-sorting exercise was conducted among Phase III MBBS students to identify topics perceived as difficult and requiring active learning. Subsequently, faculty members from the Department of Community Medicine prioritised the shortlisted topics using the nominal group technique (NGT). During the NGT process, faculty members independently rated the shortlisted topics against predefined criteria, including curriculum relevance, conceptual difficulty, suitability for active learning, applicability to public health practice, and feasibility within the available teaching schedule. The ratings were aggregated to obtain a priority ranking, which was used to select the final topics for the study. By this method, six NHP topics were selected, namely, National Tuberculosis Elimination Programme (NTEP), National AIDS Control Programme (NACP), Reproductive and Child Health (RCH) Programme, National Programme for Prevention and Control of Non-Communicable Diseases (NP-NCD), National Vector Borne Disease Control Programme - Malaria (NVBDCP - Malaria), and National Vector Borne Disease Control Programme - Other Vector-Borne Diseases (NVBDCP - Other VBDs).

The two batches were assigned to opposite teaching sequences so that every topic was taught once using TBDL or SGD-MIL across the two cohorts. Batch 1 received TBDL for NTEP, NACP, and RCH and SGD-MIL for NP-NCD, NVBDCP - Malaria, and NVBDCP - Other VBDs, while batch 2 received the reverse allocation. This counterbalanced crossover structure reduced confounding by topic difficulty and batch characteristics, although the posting batch remained the unit of assignment.

Interventions

Field Exposure

Before the classroom sessions, students visited a primary health centre, where they observed the implementation of the selected NHPs using a structured observation checklist. An interactive lecture covering all six selected topics had also been conducted previously during routine theory hours.

Team-Based Decision Learning (TBDL)

TBDL was developed as a time-feasible, decision-oriented adaptation of TBL for teaching NHPs within routine community medicine sessions. While retaining the core elements of pre-class preparation, readiness assurance, collaborative teamwork, and faculty synthesis, TBDL was specifically structured around the analysis and resolution of programme-related decision-making scenarios rather than broad content-based application exercises. The emphasis was on applying epidemiological and programme knowledge to prioritise interventions, interpret relevant programme indicators, and address implementation challenges within realistic public health contexts within the time limit of two-hour sessions without losing learner engagement.

Each TBDL session lasted two hours and comprised five sequential phases: preparation, readiness assurance, team decision-making, team defence, and faculty synthesis. Before the session, students received pre-class reading materials and/or videos to facilitate preparation. Students were organised into teams of five to six members, and the same team composition was maintained across all TBDL sessions. At the beginning of each session, students completed an Individual Readiness Assurance Test (iRAT) consisting of 10 multiple-choice questions within five minutes, followed by a Team Readiness Assurance Test (tRAT) consisting of the same 10 questions within 10 minutes. The tRAT was followed by team discussion facilitated by faculty for 20 minutes to consolidate responses and resolve areas of disagreement. During the 20-minute team decision-making phase, each team analysed a NHP scenario and identified priority actions, relevant programme indicators, and key implementation constraints. This was followed by a 20-minute team defence, during which teams justified their choices and explained why alternative actions had not been prioritised. The session concluded with 20 minutes of faculty synthesis, during which the faculty validated the reasoning, corrected misconceptions, and linked the decisions to the objectives of the respective NHP without delivering a conventional lecture.

Small Group Discussion With Mini-Interactive Lecture (SGD-MIL)

Students participated in faculty-facilitated small group discussions based on the same learning objectives as the TBDL sessions. The sessions incorporated interactive questioning, case scenarios, and focused mini-lectures to clarify concepts and reinforce understanding of programme implementation. The duration, faculty, learning objectives, and content were matched with those of the corresponding TBDL sessions.

Outcomes and measurements

Immediate learning was assessed using a 20-item structured post-test for each topic: 10 scenario-based multiple-choice questions assessing application of programme knowledge to decision-making in realistic public-health situations, five assertion-reason questions, and five multiple-response questions. Each correctly answered item was awarded one mark, with no negative marking, giving a total score range of 0-20. The post-test items were mapped to session learning objectives and reviewed by senior community medicine faculties in medical education for content validity. Assessment sheets were anonymised using study identification codes and were evaluated by a faculty member who was not involved in teaching the sessions or administering the assessments. As the post-test consisted of objectively scored items with predefined correct responses, inter-rater reliability was not applicable.

After completion of all six sessions, short-term retention was assessed using a structured viva voce with standardised questions and predefined scoring criteria out of 20; each student was assessed on one randomly selected topic from each teaching condition, producing paired observations. Depending on the position of the topic within the teaching sequence, the interval between the teaching session and the retention assessment varied, with the assessment conducted approximately seven to 14 days after the respective teaching session. The assessment used a 20-point scoring rubric comprising five domains: identification of the core programme issue; application of programme principles; public-health reasoning and justification; practical feasibility; and clarity, communication, and organisation. Each domain was assigned four marks, giving a maximum score of 20. The retention assessment was conducted as part of the routine in-posting assessment schedule. Formal inter-rater reliability was not established for the viva assessment.

​​​​Learner engagement was assessed using an adapted version of the Learner Engagement Instrument (LEI) developed by Stephenson et al. and subsequently validated by Cook and Stephenson for health professions education [9,10]. The original LEI comprises eight items assessing learner engagement across the domains of emotional, behavioural, and cognitive engagement, with responses recorded on a five-point Likert scale ranging from 1 (strongly disagree) to 5 (strongly agree). Considering the collaborative nature of TBDL, two additional items related to peer learning and collaborative interaction were incorporated into the instrument following expert review and content validation. Two peer-learning items were added to capture the perception of learning through interaction with peers and the contribution of peer discussion to understanding and application of concepts, both of which were relevant to the team-based intervention; the adapted instrument was reviewed by subject and medical education experts before use. The modified instrument therefore consisted of 10 items, each scored on a five-point Likert scale. Overall engagement scores were calculated by calculating mean scores of eight items originally included in LEI. The two additional peer-learning items were not included in the overall engagement score, as the modified 10-item instrument had not undergone formal validation, and were analysed separately as a supplementary measure.

Following all six sessions, learner satisfaction and perception were assessed using a study-developed self-administered questionnaire. Overall satisfaction was rated on a five-point Likert scale, while comparative perception items asked students to judge whether each educational attribute was better with TBDL, better with SGD-MIL, or the same in both methods (Appendices).

Bias

Potential bias included non-random assignment by posting batch, topic-related differences, carryover or testing effects, novelty effects, and instructor variation. Topic selection and crossover exposure were used to reduce confounding by topic difficulty and batch characteristics. Instructor, duration, faculty, learning objectives, and content were matched between conditions.

Sampling and study size

Convenience sampling was employed. A formal a priori sample size calculation was not performed, as the study was conducted within the constraints of the existing undergraduate clinical posting schedule. All eligible students from two consecutive Phase III MBBS posting batches were invited to participate. A total of 56 students, comprising 28 students from each batch, were enrolled.

Unit of analysis

The posting batch was the unit of assignment to the teaching sequence. For the topic-specific analysis, the individual post-test score for each NHP topic was the observational unit, with repeated observations nested within students. The student was therefore treated as the clustering unit in the mixed-effects analysis. Outcome-specific analyses included participants with available data for the respective assessment. Students who were absent from an individual teaching session or assessment were excluded from that specific analysis but remained eligible for analyses of outcomes for which data were available. Therefore, the denominator varied across individual topic-level and outcome-level analyses.

Statistical methods

Data were entered in Microsoft Excel (Microsoft Corporation, Redmond, WA) and analysed using Jamovi version 2.7.37. Julius AI (Julius AI, Inc., San Francisco, CA) was initially used to explore data visualisations based on author-specified commands from the uploaded data; the final figures were recreated by the authors using Microsoft Excel. As students contributed repeated topic-level observations, the primary immediate-learning analysis used a linear mixed-effects model with teaching method, topic, and their interaction as fixed effects and student as a random intercept. All available observations were used without zero or mean imputation. Maximum likelihood was used for likelihood-ratio comparison. Estimated marginal means provided the adjusted overall method effect; topic-specific contrasts used Holm correction. Residual diagnostics were examined. Robustness was assessed using a student-cluster bootstrap with 600 successful replications and, separately, by excluding seven observations with absolute standardised residuals >3. Retention used the Wilcoxon signed-rank test. Engagement used paired tests with Wilcoxon sensitivity analyses where needed; Holm correction was applied across five domains. Satisfaction used the paired Wilcoxon test, while perception and preference were summarised descriptively.

Ethics statement

Ethical approval was obtained from the Institutional Ethics Committee, Government Medical College, Ernakulam (IEC No. 18/26 dated 25/05/2026) before commencement of the study. Written informed consent was obtained from all participants. Participation was voluntary, and confidentiality and anonymity were maintained. The educational interventions were conducted within routine undergraduate teaching and posed no foreseeable risk.

## Results

Fifty-six Phase III MBBS students from two posting batches participated in the intervention. The available post-test dataset contained records for 55 students and 296 observed topic-level scores; missing topic scores attributable to absence were retained as missing. Engagement data were available for 54 students, and satisfaction/perception responses were available from 51 students. Short-term retention assessment was done after a two-week follow-up period.

Among 296 observed topic-level post-test scores from 55 students, the estimated marginal mean of immediate learning was 15.38 for TBDL and 14.31 for SGD-MIL, an adjusted difference of 1.07 points (95% CI: 0.53-1.61; P < 0.001). The teaching method × topic interaction was significant (likelihood-ratio χ²(5) = 21.53; P = 0.00064). After Holm adjustment, TBDL produced higher scores for NTEP (+2.80, 95% CI: 1.44-4.16; P < 0.001), NACP (+2.16, 95% CI: 0.77-3.54; P = 0.009), and RCH (+3.26, 95% CI: 1.87-4.65; P < 0.001), but not for NP-NCD, NVBDCP - Malaria, or NVBDCP - Other VBDs, as shown in Table 1. The student random-intercept variance was 0.614, residual variance was 5.514, and the intraclass correlation coefficient was 0.10.

**Table 1:** Comparison of immediate post-test performance scores obtained after each session in TBDL and SGD-MIL. The mean and standard deviations are descriptive statistics. Mean differences, 95% confidence intervals, and Holm-adjusted P-values were obtained from the linear mixed-effects model and represent TBDL minus SGD-MIL. Scores range from 0 to 20. NTEP, National Tuberculosis Elimination Programme; NACP, National AIDS Control Programme; RCH, Reproductive and Child Health; NP-NCD, National Programme for Prevention and Control of Non-Communicable Diseases; VBD, vector-borne disease; TBDL, team-based decision learning; SGD-MIL, small group discussion with mini-interactive lecture.

Topic	TBDL (n)	SGD-MIL (n)	TBDL		SGD-MIL		Mean difference	95% CI	Adjusted p-value
			Mean	SD	Mean	SD			
NTEP	27	24	17.89	1.34	15.08	1.72	2.8	1.44 to 4.16	<0.001
NACP	23	26	18.48	1.27	16.31	2.69	2.16	0.77 to 3.54	0.009
RCH	23	26	13.48	1.83	10.23	3.28	3.26	1.87 to 4.65	<0.001
NP-NCD	27	24	14.44	3.25	15.04	2.03	-0.59	-1.95 to 0.77	1.000
VBD-Malaria	23	23	15.17	2.44	15.74	1.71	-0.54	-1.97 to 0.89	1.000
VBD-Other	27	23	12.78	3.83	13.43	2.37	-0.67	-2.05 to 0.70	1.000

Robustness analyses supported the primary findings. Student-cluster bootstrap inference gave an adjusted difference of 1.07 points (95% CI: 0.61-1.48; P < 0.001) and a significant method × topic interaction (χ²(5) = 27.64; P < 0.001). Excluding the seven high-residual observations gave a difference of 1.33 points (95% CI: 0.86-1.80; P < 0.001), with the interaction remaining significant (χ²(5) = 21.14; P = 0.00076). An exploratory complete-case analysis was also conducted among the 35 students who had complete scores, attended all six topics, and completed the corresponding assessments. The mean aggregate score was 44.80 ± 5.42 for TBDL and 42.54 ± 5.27 for SGD-MIL, out of a maximum possible score of 60. The mean paired difference was 2.26 points (95% CI: 0.50-4.02; t(34) = 2.61; p = 0.013; dz = 0.44).

Among 46 paired observations, mean short-term retention scores were 10.55 ± 3.96 after TBDL and 10.63 ± 4.11 after SGD-MIL (Table 2). The paired difference was 0.08 points (95% CI: −0.88 to 1.03). Because paired differences were non-normally distributed, the Wilcoxon signed-rank test was used; no statistically significant difference was observed (W = 396, P = 0.721).

**Table 2:** Comparison of short-term retention performance scores at two weeks obtained in TBDL and SGD-MIL (n = 46). Data are presented as median (IQR). The Wilcoxon signed-rank test was used because the paired differences were non-normally distributed. W, Wilcoxon signed-rank test statistic; TBDL, team-based decision learning; SGD-MIL, small group discussion with mini-interactive lecture.

Measure	SGD-MIL (n = 46), Median (IQR)	TBDL (n = 46), Median (IQR)	Test statistic (W)	p-value
Short-term retention score	10.0 (8.0-14.0)	10.5 (7.25-14.0)	396	0.721

Student engagement and satisfaction

Paired engagement data were available for 54 students. TBDL was associated with higher emotional engagement (mean difference: 0.70, 95% CI: 0.36-1.05; Holm-adjusted P < 0.001), behavioural engagement (0.98, 95% CI: 0.35-1.60; adjusted P = 0.006), cognitive engagement in class (0.44, 95% CI: 0.16-0.72; adjusted P = 0.006), cognitive engagement outside class (0.75, 95% CI: 0.36-1.14; adjusted P = 0.001), and peer-learning engagement (0.93, 95% CI: 0.47-1.40; adjusted P < 0.001), as shown in Table 3. Cohen's dz ranged from 0.42 to 0.55. The derived normalised overall engagement score was also higher with TBDL (mean difference: 0.39 on the 0-5 scale, 95% CI: 0.21-0.57; P < 0.001; dz = 0.59). Wilcoxon sensitivity analyses for the non-normal behavioural and in-class cognitive paired differences were concordant with the paired t-test.

**Table 3:** Distribution of learner engagement scores across the two teaching-learning methods (n = 54). Paired t-tests were used to compare engagement scores between teaching methods, with Holm adjustment for multiple comparisons. P-values for the five engagement domains are Holm-adjusted for multiple comparisons. * The overall normalised engagement score was analysed separately, and its p-value was not included in the five-domain Holm correction. Cohen's dz represents the paired effect size. Wilcoxon signed-rank tests were used as sensitivity analyses where distributional assumptions were not met. LEI, Learner Engagement Instrument; TBDL, team-based decision learning; MIL, mini-interactive lecture.

LEI - Domains (n = 54)	MIL, Mean ± SD	TBDL, Mean ± SD	Difference (95% CI)	Paired t statistic	Holm-adjusted p-value	Cohen's dz
Emotional engagement	8.23 ± 1.22	8.93 ± 1.24	0.70 (0.36 to 1.05)	4.07	<0.001	0.55
Behavioural engagement	11.92 ± 2.23	12.90 ± 1.91	0.98 (0.35 to 1.60)	3.11	0.006	0.42
Cognitive engagement in class	3.93 ± 0.83	4.36 ± 0.70	0.44 (0.16 to 0.72)	3.11	0.006	0.42
Cognitive engagement outside class	8.14 ± 1.35	8.89 ± 1.20	0.75 (0.36 to 1.14)	3.84	0.001	0.52
Peer-learning engagement	7.62 ± 1.77	8.55 ± 1.39	0.93 (0.47 to 1.40)	4.02	<0.001	0.55
Overall engagement, derived (0-5) mean	3.98 ± 0.66	4.37 ± 0.58	0.39 (0.21 to 0.57)	4.35	<0.001*	0.59

Satisfaction and perception data were available from 51 respondents. The median satisfaction was 5 (IQR: 4-5) for TBDL and 4 (IQR: 3-5) for SGD-MIL; the paired Wilcoxon signed-rank test indicated higher satisfaction with TBDL (W = 72.0, P = 0.039). A score of 5 was reported by 51.0% for TBDL compared with 35.3% for SGD-MIL. Figure 2 shows the diverging bar diagram with the distribution of Likert scores in the two interventions.

**Figure 2:**
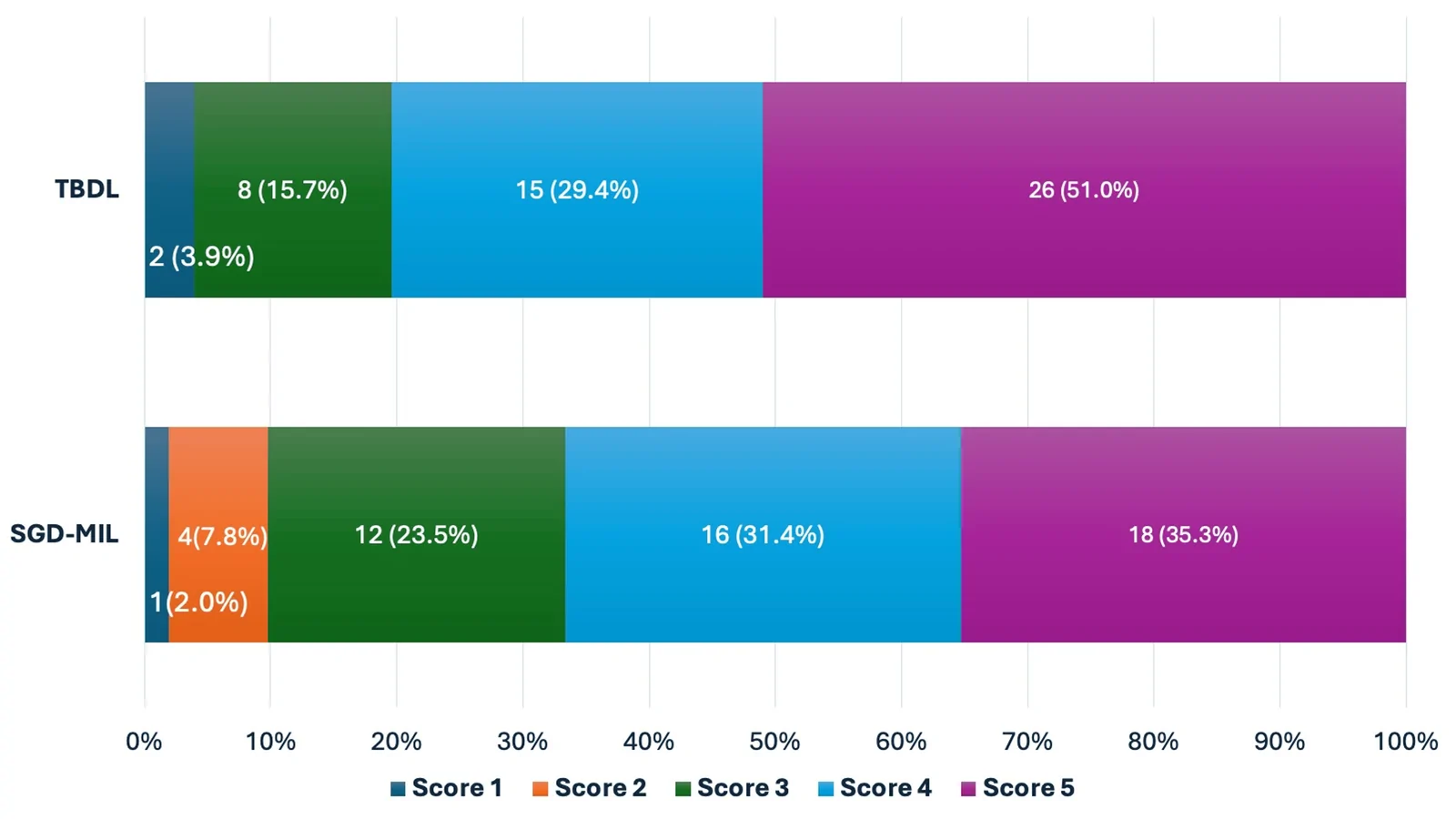
Distribution of learner satisfaction scores for TBDL and SGD-MIL (n = 51). Scores are presented as frequencies and percentages. Satisfaction was assessed using a five-point Likert scale ranging from 1 (least satisfied) to 5 (most satisfied), with higher scores indicating greater satisfaction. TBDL, team-based decision learning; SGD-MIL, small group discussion with mini-interactive lecture.

In comparative perception ratings, 54.9% favoured TBDL for application of knowledge and 52.9% for decision-making ability. The corresponding proportions favouring SGD-MIL were 31.4% for both domains, as shown in Figure 3. For specific perception statements, 80.4% agreed or strongly agreed that peer interaction was greater during TBDL, 76.5% reported greater engagement, 70.6% found TBDL more interesting, and 70.6% agreed that TBDL improved decision-making ability, as shown in the diverging bar diagram in Figure 4. At the same time, 60.8% agreed or strongly agreed that lectures helped build foundational knowledge. Overall, 56.9% preferred TBDL, 25.5% preferred both methods equally, and 17.6% preferred lectures (Figure 4).

**Figure 3:**
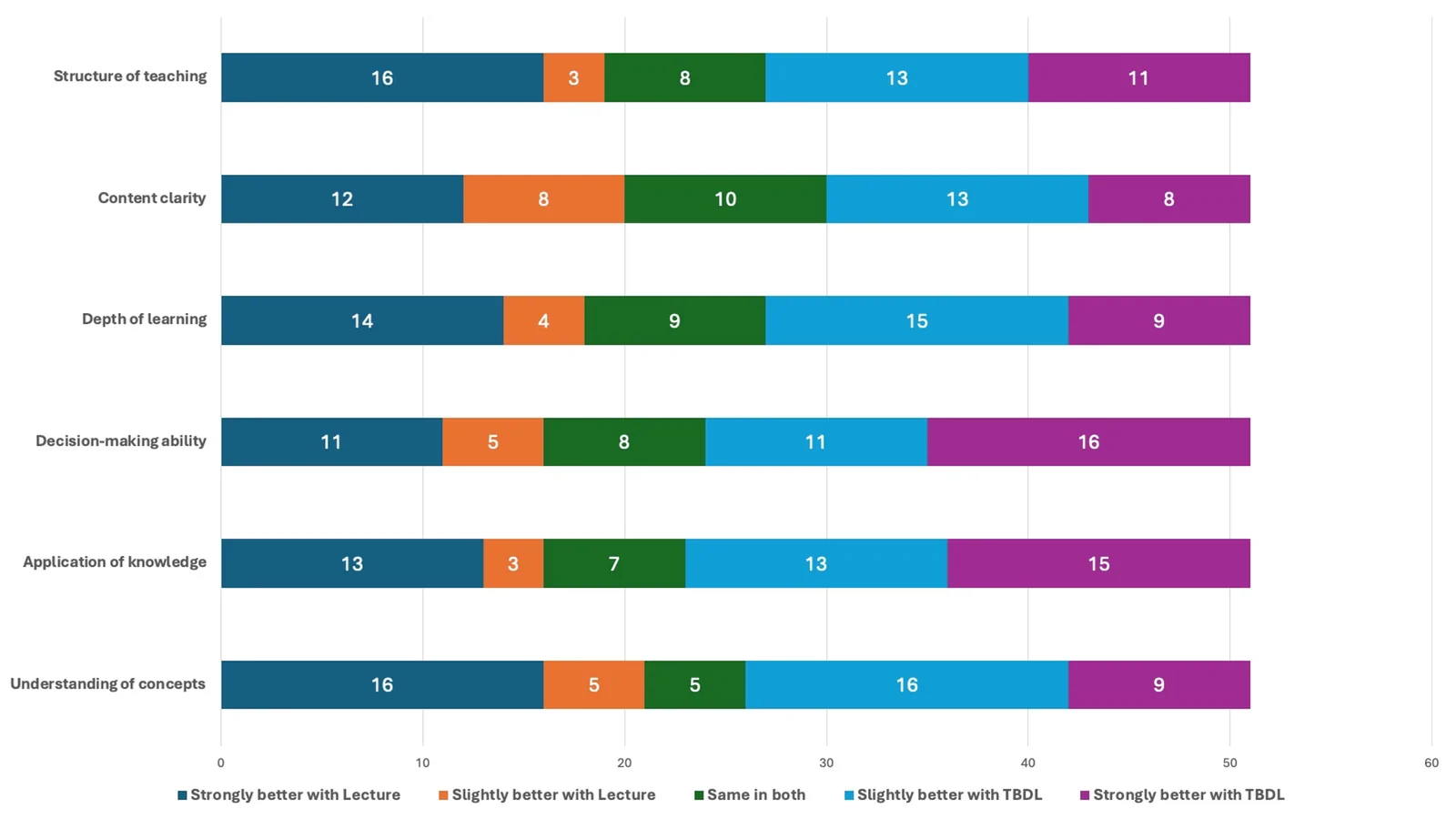
Learner perceptions of TBDL compared with SGD-MIL across various educational aspects (n = 51). Responses indicate whether learners perceived each educational domain to be better with TBDL, better with SGD-MIL, or the same with both methods. Values are presented as frequencies. TBDL, team-based decision learning; SGD-MIL, small group discussion with mini-interactive lecture.

**Figure 4:**
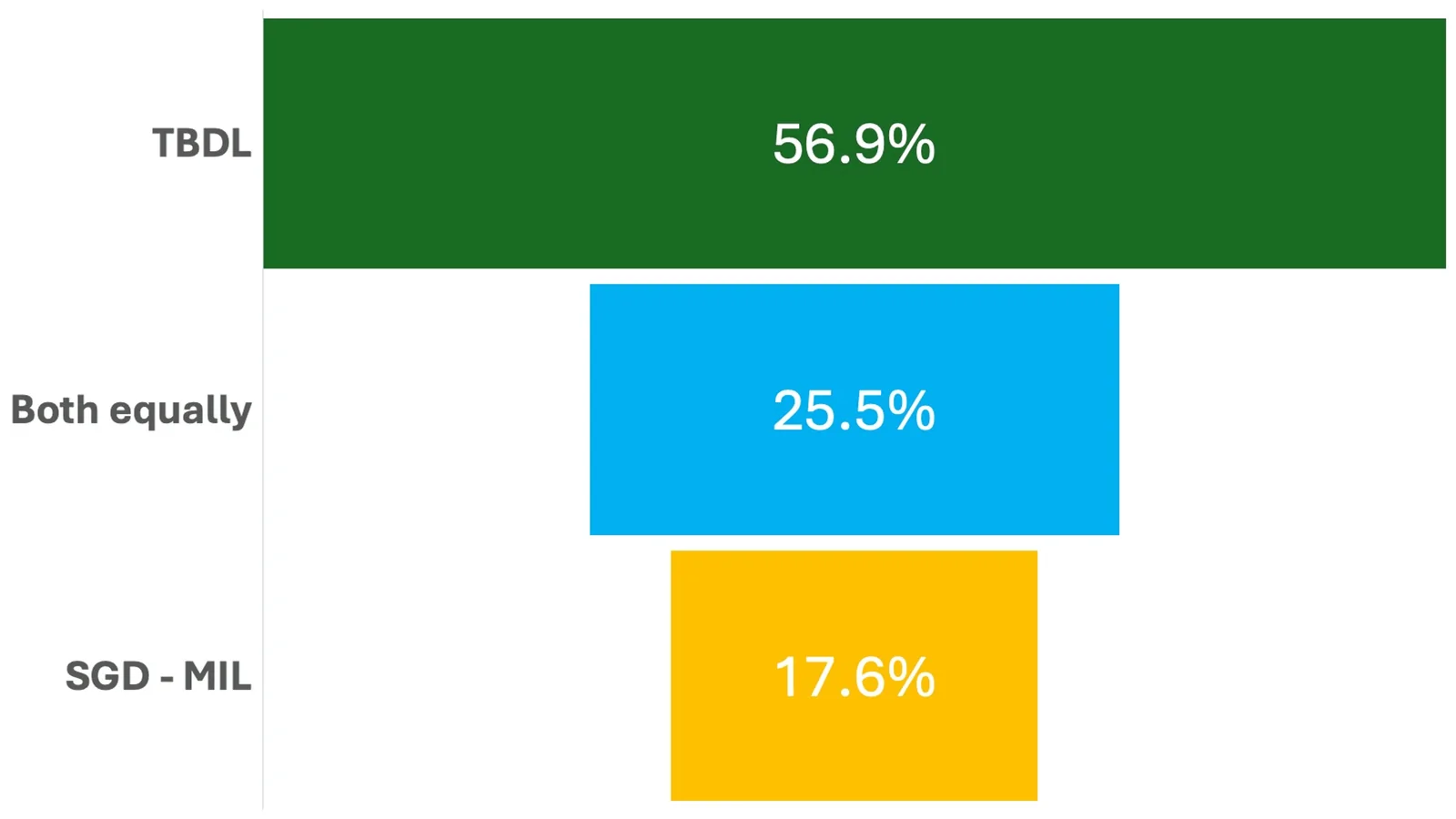
Overall student preference for teaching method (n = 51). Overall preference was recorded as TBDL, SGD-MIL, or both methods equally. Values are presented as percentages of respondents. TBDL, team-based decision learning; SGD-MIL, small group discussion with mini-interactive lecture.

## Discussion

The quasi-experimental crossover study found better immediate learning with TBDL after accounting for topic and repeated observations within students, with an adjusted mean difference of 1.07 marks (95% CI: 0.53-1.61; P < 0.001). The advantage was concentrated in NTEP, NACP, and RCH, whereas no significant differences were observed for NP-NCD, NVBDCP - Malaria, or NVBDCP - Other VBDs. Short-term retention did not differ significantly between methods, indicating that the observed benefit was primarily in immediate, topic-specific learning and engagement.

This pattern of benefit suggests that a decision-oriented adaptation of team-based learning may better align community medicine teaching with the NMC’s CBME goals. This aligns with broader literature available: a systematic review of 33 studies reported large effects favouring TBL in post-test scores (standardised mean difference (SMD) ≈ 0.96), retention (SMD ≈ 1.03), and even engagement (SMD ≈ 2.26) [11]. Another meta-analysis of 13 medical education studies (n = 1545) in China found significantly higher exam scores under TBL (SMD ≈ 2.46) compared to lecture-based learning [12]. Our finding of higher post-test scores with TBDL extends these international results into the context of Indian community medicine, reinforcing that decision-oriented collaboration improves cognitive outcomes. These findings are also consistent with the study by Jagadevi et al. done in the Indian context, which reported better post-test performance following small group discussion compared with interactive lectures among undergraduate medical students [13]. The absence of a retention difference in the current study suggests that the immediate advantage of TBDL did not translate into a measurable advantage at seven to 14 days, consistent with the mixed evidence on retention in the TBL literature. Sterpu et al. noted that while engagement and satisfaction increased with TBL, longer-term knowledge retention outcomes were inconsistent [14]. This may also reflect our short follow-up window and the use of discussion, questioning, and case-based learning in SGD-MIL.

The strong engagement findings are plausible because TBDL required preparation, collective decision-making, defence of reasoning, and peer/faculty challenge. These features promote accountability and social interaction [5,6]. TBDL outperformed SGD-MIL across all engagement domains, a notable result since engaged students tend to learn more deeply and retain knowledge better; this corroborates Xie et al.'s very large pooled engagement effect for TBL (SMD ≈ 2.26) [11]. Learner-reported satisfaction and perceptions also favoured TBDL for engagement, peer interaction, and decision-making, while recognising a complementary role for lectures in foundational knowledge. Students particularly gave stronger responses for the decision-making focus of TBDL; they felt better able to “prioritise interventions” and “apply concepts” after TBDL exercises. These findings are consistent with Shivanand et al., who reported that 70% of students strongly agreed that TBL significantly enhanced their understanding of the topic and 65% of students strongly agreed that the method improved active participation [15]. Scientific evidence in the literature on students' perceptions of higher-order cognitive skills like prioritisation and application remains limited.

Although these findings are encouraging, they should, however, be interpreted in the light of the methodological limitations of the study design, sample, and setting. The study was conducted at a single medical college with a small convenience sample. Assignment by posting batch rather than individual randomisation may have introduced confounding by batch, topic, and period. Although the counterbalanced design enabled both batches to experience both teaching methods across different topics, only two intact posting batches were included, limiting the ability to adjust the teaching-method effects from batch or session-level variation. While TBDL showed higher scores than SGD-MIL in both batches, statistically significant topic-specific differences were observed only in one batch. The limited number of batches and students might have therefore reduced the ability to detect consistent effects across batches. Some topic-level observations were missing due to absence; although the mixed model retained available data without imputation, missing-data bias cannot be excluded. Satisfaction and perceptions were learner-reported using a study-developed questionnaire. Facilitator and novelty effects may also have influenced the observed differences. Finally, outcomes were limited to immediate learning and short-term retention, while long-term retention and sustained effects of the intervention were not evaluated. Therefore, the findings should be interpreted as evidence of an association with immediate learning and learner experience rather than definitive evidence of superiority or sustained educational benefit. Despite these limitations, the intervention may be transferable to other undergraduate health-professions settings where learners must apply public health programme knowledge to real-world decisions. Replication with multiple institutions, instructors, cohorts, and longer follow-up is needed before broad generalisation. Replication across multiple institutions, instructors, and cohorts, with longer follow-up, is warranted before broader generalisation.

These considerations also indicate several priorities for future research. TBDL is an adapted form of TBL focused on public health strategy and could be applied to other topics (e.g., outbreak investigation and health promotion) without sacrificing content coverage. Future research should use multi-centre randomised or cluster-randomised designs, assess long-term retention and transfer to practice, evaluate faculty workload, and standardise and test TBDL across instructors, institutions, and learner groups.

## Conclusions

TBDL, a decision-oriented adaptation of team-based learning for public health education, was associated with better immediate learning for three of six NHP topics, along with higher learner engagement and satisfaction compared with SGD-MIL among Phase III MBBS students. However, the benefit in immediate learning was evident for three NHP topics, which was not reflected in short-term retention, as the scores obtained showed no statistically significant difference between the two approaches. These findings suggest that incorporating structured preparation, team-based decision-making, defence of reasoning, and faculty synthesis into community medicine teaching can provide learners with greater opportunities to apply knowledge and engage collaboratively with public health problems.

TBDL may therefore serve as a complementary active learning strategy within competency-based undergraduate medical education, particularly for NHP topics that require prioritisation, application of knowledge, and decision-making rather than factual recall alone. Given the limited number of posting batches and the potential for batch- and session-level influences, these findings should be interpreted cautiously. Its use should be considered alongside other established teaching approaches, with further multi-centre studies involving larger cohorts, different instructors, and longer follow-up needed to determine its wider applicability and sustained educational impact.

## Appendices

Appendix 1 - Learner Engagement Instrument (LEI)

Eight-item validated scale

Five-point Likert scale (Strongly Disagree → Strongly Agree)

Covers emotional + behavioural + cognitive engagement

Instructions to Students

Please indicate your level of agreement with the following statements regarding the session:

Scale: 1 = Strongly Disagree, 2 = Disagree, 3 = Neutral, 4 = Agree, 5 = Strongly Agree

LEI Items

**Table 4:** Original Learner Engagement Instrument (LEI) items and additional items for social/team engagement. Items 1-8 represent the original validated LEI items and were used for the calculation of the overall engagement score [9]. The additional social/team engagement items were developed for the present study to capture peer learning and collaborative interaction relevant to team-based decision learning (TBDL). * These additional items were not part of the original LEI and were not included in the overall engagement score. All items were rated on a five-point Likert scale ranging from 1 (Strongly Disagree) to 5 (Strongly Agree). Source: Adapted from Stephenson et al. [9] under the terms of the Creative Commons CC BY license.

LEI items	1	2	3	4	5
Emotional engagement					
I enjoyed this session					
I was interested in this session					
Behavioural engagement					
I actively participated in this session					
I avoided distractions during this session					
I was actively involved in learning					
Cognitive engagement (In-session)					
I was fully absorbed in this session					
Cognitive engagement (Transfer/Motivation)					
I will apply what I learned to my practice					
I am motivated to learn more about these topics					
Social/team engagement (Not in original LEI)*					
I learned from my peers during the session					
Group discussion improved my understanding					

**Table 5:** Items belonging to each domain as per the original Learner Engagement Instrument.

Domain	Items
Emotional	1, 2
Behavioural	3, 4, 5
Cognitive	6
Transfer/Motivation	7, 8

Appendix 2 - Sample scenarios with questions used for discussion in the team-based decision learning (TBDL) approach

National Tuberculosis Elimination Programme (NTEP)

Scenario 1: Delayed Diagnosis in High-Risk Patient

A 55-year-old male with poorly controlled diabetes presents to your primary health centre (PHC) with:

Cough for three weeks

Weight loss

Intermittent fever

He visited a private practitioner twice and was given antibiotics without relief. He is a daily wage worker and reluctant to miss work.

Decision Questions

1. What is your MOST appropriate initial diagnostic approach under NTEP?

2. How will you ensure early diagnosis without losing the patient to follow-up?

3. What public health action will you initiate at this stage (before confirmation)?

Scenario 2: Treatment Initiation & Adherence Challenges

Cartridge-based nucleic acid amplification test (CBNAAT) confirms pulmonary tuberculosis (TB) (rifampicin-sensitive).

The patient lives 15 km away, works daily, and says: “I cannot come frequently to the hospital.”

Decision Questions

1. What treatment regimen will you initiate under NTEP?

2. How will you ensure treatment adherence in this patient? Justify your choice based on feasibility.

3. What social support mechanisms can you activate? Justify the importance in treatment success.

National AIDS Control Programme (NACP)

Scenario 1: Behaviour Change Communication (General Population)

You are the Medical Officer of a PHC in a semi-urban area.

Recent data show:

• Increasing HIV positivity among young adults (20-30 years)

• Low condom use

• Poor awareness about HIV transmission

Decision Questions

1. What behavioural intervention strategy will you prioritise at the community level?

- Mass information, education, and communication (IEC) vs. focused behaviour change communication (BCC) vs. school-based intervention

- Justify based on NACP behaviour change strategies.

2. Which communication channels will you use for maximum impact in this population?

- Social media, peer groups, Accredited Social Health Activist (ASHA)-led sessions

- Justify based on target audience characteristics.

3. How will you evaluate the effectiveness of your intervention?

- Knowledge surveys, condom uptake, testing rates

- Justify using programme indicators.

Scenario 2: Stigma, Adherence & Social Support

A 32-year-old woman on antiretroviral therapy (ART) for six months is irregular with treatment.

On probing, she reveals:

• Fear of disclosure at home

• Financial difficulties

• Depression symptoms

Decision Questions

1. What is your primary approach to improve adherence?

- Strict instruction vs. counselling vs. social support

- Justify using a patient-centred care approach.

2. What support systems can you mobilise under NACP?

- Link ART centres, non-governmental organisations (NGOs), support groups

- Justify based on programme support structure.

3. How will you address mental health and stigma in this patient?

- Referral, counselling, confidentiality

- Justify using holistic care principles.

Reproductive and Child Health Programme - Reproductive, Maternal, Newborn, Child, Adolescent Health and Nutrition (RMNCHA+N) 

Scenario 1: Late ANC Registration With Risk Factors

A 24-year-old pregnant woman (G2P1) comes to your PHC for the first time at 24 weeks of gestation. She reports:

No prior antenatal care (ANC) visits

History of anaemia in previous pregnancy

Lives in a remote area with limited transport

On examination: Haemoglobin (Hb) estimated to be clinically low.

Decision Questions

1. What immediate package of services will you provide during this visit? Justify based on ANC guidelines under Reproductive, Maternal, Newborn, Child, and Adolescent Health (RMNCH+A).

2. How will you classify and manage her risk status? Decide whether she is high-risk and justify referral vs. PHC-level care.

3. What strategies will you use to ensure continuity of care till delivery? Justify using a continuum of care approach.

Scenario 2: Decision on Place of Delivery

A 19-year-old primigravida in a rural area expresses a preference for home delivery due to family tradition. She is currently 36 weeks pregnant, with no complications so far.

Decision Questions

1. What is your decision regarding place of delivery? Justify based on RMNCH+A and safe motherhood policies.

2. How will you counsel the patient and family effectively? Justify using IEC/BCC principles.

3. What government schemes can you use to support your decision? Justify how they reduce barriers.

National Programme for Control of Non-Communicable Diseases (NP-NCD)

**Scenario 1** - **"Why Are Our Screening Numbers Not Translating Into Control?"**

You are the Medical Officer of a PHC serving 35,000 population.

During district review, your PHC is praised because:

Ninety percent of eligible adults (>30 years) have undergone population-based screening.

More than 4,000 people are registered on the Non-Communicable Diseases (NCD) portal.

However:

Only 18% of hypertensive patients have controlled blood pressure (BP).

Only 12% of diabetic patients have controlled blood sugar.

Nearly half of registered patients have not visited the PHC in the last six months.

The District Programme Officer reminds you that the district is expected to contribute to the national "75 by 25" initiative.

You have limited manpower and cannot conduct more screening camps.

Decision Questions

1. What should be your immediate priority-more screening or strengthening follow-up? Justify.

2. How would you use ASHAs, Auxiliary Nurse Midwives (ANMs) and Ayushman Arogya Mandirs to improve control rates?

3. Which indicators would you monitor over the next six months to determine success?

Scenario 2 - "The Silent Failure of Cancer Screening"

Your PHC has achieved the annual target for cervical cancer screening.

Review of records shows:

A total of 320 women were screened by visual inspection with acetic acid (VIA), and 24 VIA-positive women were identified.

When the district team audits the programme:

Only eight women reached the referral centre, 10 women refused referral, and six women could not be traced. One year later, two women present with advanced cervical cancer. District authorities ask for an explanation.

Decision Questions

1. What is the most important programme failure in this situation?

2. How should referral tracking be strengthened?

3. Should future programme evaluation focus on screening numbers or completion of referral pathway? Defend your answer.

National Vector Borne Disease Control Programme - Malaria (NVBDCP - Malaria)

Scenario 1 - Fever in a Migrant Labour Camp

You are the Medical Officer of a PHC in Ernakulam district.

During routine surveillance, an ASHA reports that five migrant workers from Odisha residing in a labour camp have developed fever over the last week. One worker tests positive for malaria by rapid diagnostic test (RDT) at a private laboratory. The labour camp houses over 120 workers from different endemic states. Most workers continue attending work despite fever. The Panchayat President is worried that local transmission may occur.

Decision Questions

1. What should be your immediate public health response?

2. Should surveillance be limited to symptomatic individuals, or should additional screening be conducted? Justify.

3. What measures are required to prevent re-establishment of malaria transmission in this area?

Scenario 2 - The Negative RDT Problem

A 32-year-old plantation worker from Attappady presents with high fever, chills and sweating. The RDT performed at the PHC is negative. However, the patient recently returned from a malaria-endemic district in Chhattisgarh. Clinical suspicion remains high. The nearest microscopy centre is 30 km away. Junior staff suggest sending the patient home with symptomatic treatment.

Decision Questions

1. What should be the next diagnostic step?

2. Should programme action depend solely on the RDT result?

3. What surveillance records and reporting mechanisms should be initiated immediately?

National Vector Borne Disease Control Programme - Other Vector Borne Diseases (NVBDCP - Other VBDs)

Scenario 1 - "The Fever that Became Shock"

You are the Medical Officer of a Family Health Centre in Ernakulam. A 19-year-old engineering student presents on the fifth day of fever. His fever has subsided since morning, but he now complains of:

- Persistent vomiting

- Severe abdominal pain

- Extreme weakness

The following were the findings during examination:

- Cold extremities +

- Capillary refill time >3 seconds

- Pulse - rapid and weak

- BP - 100/85 mmHg

The casualty medical officer feels that the patient is improving because the fever has disappeared and suggests observation in the OPD. Meanwhile, two other similar cases have been admitted from the same ward during the previous week.

Decision Questions

1. What stage of dengue illness is this patient likely experiencing?

2. What should be your immediate clinical and referral decision?

3. Beyond managing this patient, what public health actions should the PHC initiate in the affected ward?

Scenario 2 - "Six Cases in One Ward"

During weekly surveillance review, your Junior Health Inspector (JHI) reports:

- Six fever cases from a single municipal ward

- One laboratory-confirmed dengue case

- All cases occurred within seven days

- Population of the ward approximately 11,000

The Municipal Councillor demands immediate fogging throughout the municipality. The Health Inspector argues that source reduction should be prioritised. Resources are limited, and only one rapid response team is available.

Decision Questions

1. Does this situation qualify as an outbreak under programme definitions?

2. What actions should be initiated within the first 24 hours?

3. If resources are limited, how would you prioritise source reduction, larval control, and fogging?
